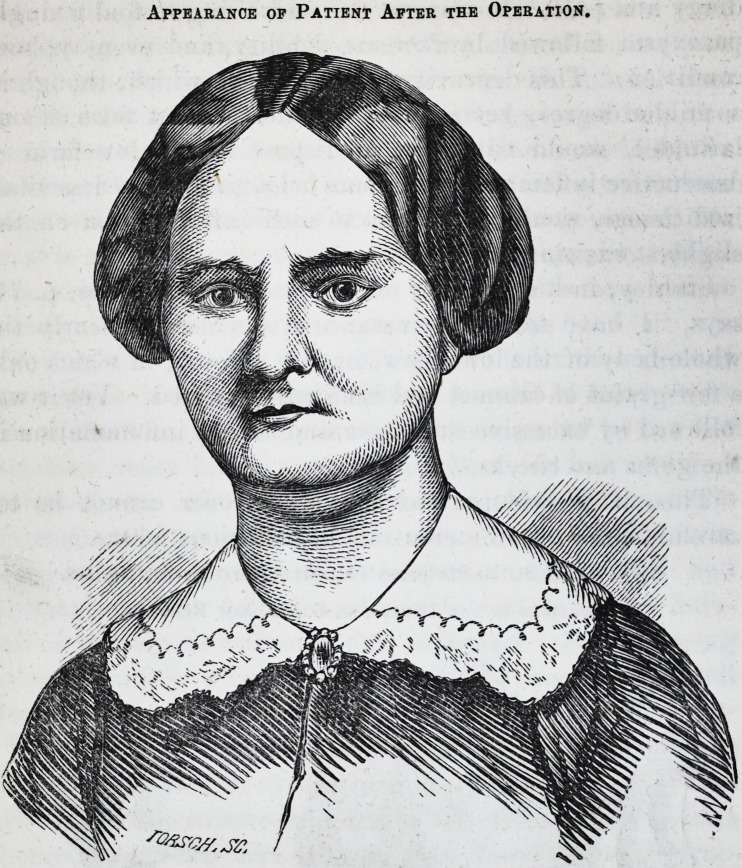# Case of Necrosis of the Upper and Lower Jaw and Operation

**Published:** 1859-04

**Authors:** G. Grant

**Affiliations:** Newark, N. J.


### ARTICLE X.

### *Case of Necrosis of the Upper and Lower Jaw and Operation.*

BY G. GRANT, M. D. of NEWARK, N. J.

MRS. B. of this city, æt. 26, of good constitution, excellent health and physique, about the 1st Sept. 1853, was attacked with intermittent fever, which disease prevails during a larger period of the year in that part of the city in which she resided. The history of the disease in her case did not differ from the ordinary course of the simpler varieties of periodical fever. Her medical attendant during the course of the disease administered a few grains of calomel, when in a very short time inflammation of the mucous membrane of the mouth occurred, which extending through the subjacent tissue to the periosteum of the upper and lower jaw, resulted in necrosis of nearly the whole of these two bones, with its accompanying evils of fetid discharges, fistulous abscesses, &c., and soon resulting in a general cachectic condition of the system. This condition continued after the paroxysms of the fever had disappeared, and during the convalescence—a period of several months. In the early part of 1854, I was consulted by her, and on examination, found that the diseased portion involved nearly the whole of the upper jaw of the right side, a considerable portion of the left side, together with a part of the lower jaw. The condition of the patient at this time, was indeed distressing; a foul sanious discharge impregnating her food, emitted an almost intolerable fetor. Advised the removal of the entire diseased portion of bone, to which, after several months delay, she consented. A wash of dilute muriatic acid in the course of a few days, entirely removed the odor.

On the 12th August, assisted by Dr. Milton Baldwin, I removed the portion of the upper jaw diseased. The incisions were made within the mouth on either side of the bone, and carried up until the characteristic feeling of resistance indicated the presence of hard living bone. The diseased portion was then pried off and removed. About a week subsequently I removed the portion of the lower jaw, assisted by the same gentleman. The operation was performed in the same manner. Such was its extent, that upon detaching the bone it became necessary to saw it in two in order to remove it from the mouth. There was little or no hemorrhage during or subsequent to either operation. There was no employment at any time of any anæsthetic agent to produce insensibility. The patient speedily rallied from the nervous shock, and in a few weeks had almost entirely regained her usual vigor and health.

This case derives its interest not only from the extent of the necrosis involving such a large amount of bony structure of the face, but from the thought suggested by the fact that the disease had its origin during an attack of intermittent fever. Calomel at any time is a poison to patients of certain idiosyncrasy, but such idiosyncrasy could not be said to exist in my patient, inasmuch as both before and subsequent to the operation, she had been under the influence of this agent without being salivated. It was during the fever, and by a very few grains of the drug that its evil effect was manifested.

It is a proper subject of inquiry whether, in intermittent fever, there is not a dyscrasia of the blood caused by the miasm which predisposes the system to the injurious effects of certain remedies like calomel, and would favor destructive inflammation in those tissues which are the least highly organized. This depravity of the system induced by the malarious poison, is very evident in residents of the tropics, where the intensity of the fever gives to all its symptoms a more marked character. From observations made during nearly a year's residence at the Isthmus of Panama, I found this fact constantly and uniformly marked. The fever is designated as *calentura*, and differed only from intermittent fever of more temperate latitudes in the greater intensity of all the symptoms and the excessive anæmia attendant upon it. The blood seemed to lose nearly all its coloring matter, the gums, lips and skin became dusky, dingy and pale. It was no unusual thing to find a single paroxysm followed by extreme debility, and even typhoid condition. This depravity of the system, which, though in a milder degree, yet follows the intermittent fever of our latitudes, would naturally predispose to the low form of destructive inflammation. Bones belonging to the less vitalized tissues, would be subject to such inflammation on the slightest exciting cause.

Stanley, in his Treatise on Diseases of the Bones, p. 77, says, "I have seen* an instance of necrosis in nearly the whole body of the lower jaw, in case of fever, in which only a few grains of calomel had been administered. Yet it was followed by excessive salivation and severe inflammation in the gums and cheeks."

Thus it is evident that the practitioner cannot be too careful in the use of mercurials in malarious fevers.

* Museum of St. Bartholomew's Hospital, First Series, No. 102.

PORTIONS OF THE UPPER AND LOWER JAW REMOVED.

APPEARANCE OF PATIENT AFTER THE OPERATION.

MESSRS. EDITORS:

The above interesting case, taken from the *Transactions of the New Jersey State Medical Society*, came under my observation before the operation performed by Dr. Grant, who, allow me here to say, deserves great credit for the able and successful treatment of this distressing case. Mrs. B. stated that before she was taken sick, she was not aware that she had an unsound tooth in her head. She was but lately married, healthy, well featured, in fact comely in appearance, but when we saw her, after her recovery from the attack of fever by which she had been prostrated, she presented truly a lamentable appearance. If we recollect aright, she had lost nearly or all of her teeth, and the process of both upper and under maxillary, was entirely denuded of the surrounding membranes, and the fleshy tissues were in such a state of suppuration that the fœtor arising from the parts was perfectly unbearable, so much so, as to sicken any one who remained near her for any length of time. Her countenance was pallid, and her face so deformed by the sinking in of the facial muscles, that she presented the appearance of a person of three score years and ten, rather than that of a young woman of twenty-six.

A few weeks since she called upon us, and we were gratified to find that since the operation, the parts have become perfectly restored, minus the bones. Her voice, owing to the loss of the palatine arch and its surroundings, is a little old womanish. Her general health good. She has for some time used an artificial set of teeth, on platina plate, made by Mr. James Fowler, dentist, of this city, who has been so successful in adapting the case to the parts, that she readily masticates and articulates without difficulty. The case reported, clearly illustrates the direful effects of calomel, although in small doses, and is also interesting to the dental surgeon, insomuch (as after such an operation) the successful adaptation of a dental operation so as to, in a great measure, supply the loss of the natural organs, should encourage members of our profession, to undertake to produce the like result, should an opportunity offer.

Respectfully, yours,

*Newark, N. J.* G. F. J. COLBURN.

## Figures and Tables

**Figure f1:**
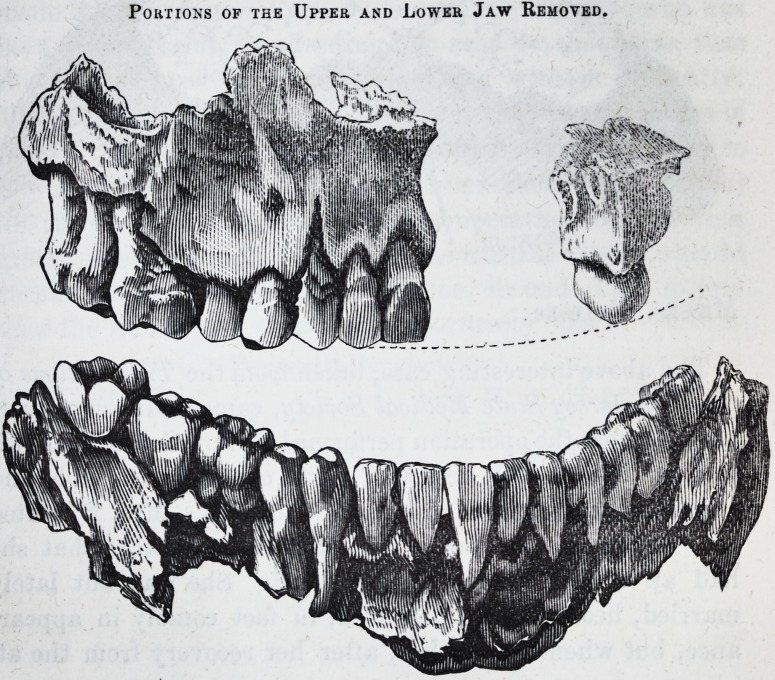


**Figure f2:**